# Isolated Sub-Pulmonic Valve Endocarditis in a Patient With a History of Konno Procedure and Mechanical Aortic Valve

**DOI:** 10.7759/cureus.17594

**Published:** 2021-08-31

**Authors:** Jacob Alex, Harshil Patel, Roshni Shah, Souheil Saba, Marcel Zughaib

**Affiliations:** 1 Department of Internal Medicine, Ascension Providence Hospital, Southfield, USA; 2 Department of Cardiovascular Medicine, Ascension Providence Hospital, Southfield, USA

**Keywords:** trans-thoracic echocardiogram, modified konno procedure, endocarditis, sub-pulmonic valve, ross-konno procedure

## Abstract

Pulmonic and sub-pulmonic valve endocarditis are rarely encountered in clinical practice. We present the first case of isolated sub-pulmonic endocarditis. A 30-year-old man with a history of mechanical aortic valve presented to the emergency department with multiple complaints including nausea, vomiting, body aches, and fevers. The patient underwent surgical resection for sub-aortic stenosis followed by a modified Konno procedure later in life. A modified basal short-axis view on the trans-thoracic echocardiogram revealed a sub-pulmonic mobile structure highly suggestive of infective endocarditis. Blood cultures grew methicillin-sensitive *Staphylococcus aureus* within 24 hours. Higher oxygen demand prompted chest imaging, chest CT showed the development of bilateral airspace consolidation, suggestive of pneumonia. After treatment with extended intravenous antibiotics, follow-up echocardiogram four months later showed no identifiable sub-pulmonic vegetation.

This case describes a situation where clinicians may suspect infective endocarditis in a typical location such as a mechanical aortic valve. However, in patients who develop pneumonia, infective endocarditis of the right heart should be suspected. The pulmonic valve and sub-pulmonic ridge are often difficult to image given their anatomical location, a modified basal short-axis view on trans-thoracic echocardiogram can better image these structures.

## Introduction

Infective endocarditis (IE) of the right heart involving exclusively the pulmonary valve is a rare phenomenon with an estimated prevalence of approximately 2% of all IE cases [1]. To our knowledge, there are no reported cases of sub-pulmonic valve endocarditis. The diagnosis could be challenging in patients with a mechanical aortic valve, which could be perceived as the nidus of infection. We present a case of a 30-year-old male with a history of complicated cardiac surgeries in his childhood and the presence of a mechanical aortic valve who developed sub-pulmonic valve endocarditis.

## Case presentation

**Figure 1:**
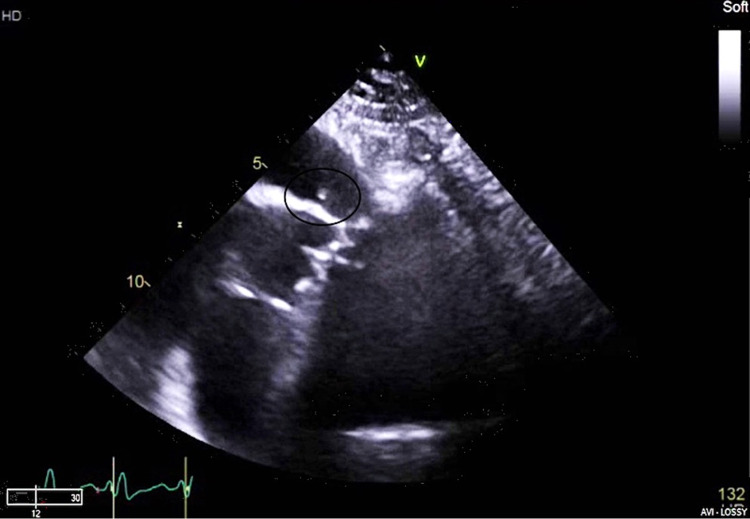
Trans-thoracic echocardiogram modified basal short-axis view showing a mobile 5 mm vegetation on the sub-pulmonic ridge.

A 30-year-old male with a normally functioning mechanical aortic valve and non-compliance to warfarin therapy presented to the emergency department with complaints of fever, chills, nausea, vomiting, headache, and body aches which started abruptly the day before. The patient denied use of recreational intravenous drugs, recent dental procedures, or previous infective endocarditis. On admission, he had a body temperature of 101.9°F, a heart rate of 76 beats/min, a respiratory rate of 18 breaths/min, and blood pressure of 145/68 mmHg. The physical examination was notable for grade 4/6 holosystolic murmur heard throughout the precordium with an audible click, without any evidence of immunologic or embolic phenomenon. Laboratory workup was notable for white blood cell count of 15,070 per microliter of blood. He met 3/4 systemic inflammatory response syndrome (SIRS) criteria. A trans-thoracic echocardiogram (TTE) revealed a normally functioning mechanical aortic valve without any evidence of endocarditis, preserved left ventricular ejection fraction, and moderate pulmonic valve stenosis with a peak velocity of 3.8 m/s and peak gradient of 58 mmHg across the pulmonic valve. There was no evidence of pulmonic valve or sub-pulmonic valve vegetation. The patient was empirically started on broad-spectrum antibiotics. The blood culture grew methicillin-sensitive *Staphylococcus aureus* (MSSA) within 24 hours, repeat blood cultures on days two and four of admission also grew MSSA. On day four of admission, the patient had increasing oxygen requirements and maximum body temperature of 103.5°F, without associated cough or sputum production. This prompted a chest x-ray, which revealed a new right basilar opacity, not present on the chest x-ray from day one. A CT scan of the chest revealed a dense airspace consolidation in the right lower lobe with additional heterogeneous left lower lobe opacities. These radiological and clinical findings were suggestive of pneumonia, which raised suspicion of right-sided endocarditis having caused septic embolization to the lungs. A trans-esophageal echocardiogram (TEE) was performed which confirmed moderate pulmonic valve stenosis as noted on the TTE. However, vegetation on right-sided valves was not detected on TEE despite efforts. Another attempt using a modified basal short-axis view on a trans-thoracic echocardiogram revealed a hyperechoic mobile 5 mm vegetation on the sub-pulmonic ridge (Figure 1 and Video 1). Intravenous antibiotics were continued and the cardiothoracic surgery team was consulted.

**Video 1:** Mobile mass at the sub-pulmonic ridge viewed on trans-thoracic echocardiogram modified basal short-axis view.

A review of operative notes from mechanical aortic valve implantation surgery revealed that discrete sub-aortic stenosis was surgically resected in his childhood. This was followed by a recurrence of a more complex form of sub-aortic stenosis, treated by a modified Konno procedure and repair of a mild supravalvular aortic stenosis with a glutaraldehyde soaked bovine pericardial patch. Over the subsequent years, the patient developed progressively worsening aortic stenosis with aortic insufficiency and left ventricular hypertrophy, which led to a mechanical aortic valve replacement at the age of 15 years. He received a 27 mm St. Jude aortic valve (St. Jude Medical, Saint Paul, MN), along with an aorto-ventriculoplasty with a Dacron patch constructed from a 24 mm diameter Hemashield Dacron tube graft (St. Jude Medical, Saint Paul, MN). The right ventricle and right ventricular outflow tract appeared normal intra-operatively. The operative records of his prior surgeries were not available to review. The patient was deemed not to be a surgical candidate because of his history of complex cardiac surgeries. He was discharged on intravenous antibiotics for a total duration of six weeks and warfarin was restarted. After completion of antibiotic therapy, a follow-up echocardiogram four months later showed resolution of sub-pulmonic valve endocarditis with no vegetations identified.

## Discussion

Infective endocarditis of the right-sided valves is less common than on the left. Within this group, isolated pulmonic valve endocarditis accounts for approximately 2% of all IE cases [1]. A review by Chowdhury and Moukarbel reported only 70 published cases of isolated pulmonic valve endocarditis between 1979 and 2013 [2]. The lower incidence of right-sided IE can be attributed to lower blood flow jet velocities and pressure gradients causing insignificant mechanical stress on the endocardium, lower oxygen saturation in venous blood, and a lower prevalence of congenital malformations in the right heart [1,3]. Right-sided IE is more frequently encountered in males, intravenous drug users, alcoholics, patients with chronic intravenous catheters or intra-cardiac devices, immunosuppression, and congenital heart disease [3,4].

The patient was noted to have moderate pulmonic valve stenosis on echocardiography. Based on the fact that this finding was not present intra-operatively during previous cardiac surgery, it could be hypothesized that the patient developed pulmonic valve stenosis as a complication of the prior surgery. The stenotic pulmonic valve may have provided a substrate to form vegetation on the sub-pulmonic valve structures due to a relative stagnation of blood in the right ventricular outflow tract. Although the initial clinical presentation led to a suspicion of aortic valve endocarditis due to the presence of a mechanical aortic valve, the development of pneumonia provided a valuable hint to suspect right-sided endocarditis. Standard TEE and TTE views did not reveal any vegetation, a modified short-axis view at the base on a TTE helped uncover the presence of mobile vegetation on the sub-pulmonic ridge in the right ventricular outflow tract. An epidemiological survey by Hoen et al. revealed 68% of patients with right-sided IE had septic pulmonary embolism [5]. Septic pulmonary emboli often present with fever, dyspnea, pleuritic chest pain, and hemoptysis, of which our patient had only fever. The findings on chest x-ray are usually nonspecific and a CT scan is more useful in identifying the characteristic peripheral cavitary lesions [6]. Our patient however did not have these characteristic cavitary lesions in the lungs on CT scan, but had opacities consistent with pneumonia. Unlike the presented case, a TEE provides an unobstructed view of the intra-cardiac structures and has a higher diagnostic yield for identifying pulmonic valve endocarditis [7,8]. Intuitively, a TTE could be thought of as a better imaging modality for the right heart structures due to its proximity to the anterior chest wall. However, in adults, it may be challenging to get a cross-sectional view of the pulmonic valve with TTE. Modification during imaging could help better visualize the pulmonic valve. 

The modified Konno operation is performed mainly in the pediatric population with multi-level left ventricular outflow tract obstruction using the pulmonic valve as an autograft to repair the stenosis. This procedure is seen as a last resort with an early mortality rate of 12.5% [9]. The literature is limited with regard to the long-term complications of this procedure. The authors did not have an operative record of patient’s prior surgeries, viz. the aortic stenosis resection and the modified modified Konno procedure, which is a potential limitation of this presented case. There was no mention of any structural abnormality of the pulmonic valve or right ventricular outflow tract (RVOT) in the operative report of mechanical aortic valve implantation, which leads to skepticism of whether the pulmonic valve was manipulated during prior surgeries. If, however, the pulmonic valve was indeed utilized as an autograft as is usually performed during the modified Konno procedure, the manipulation of the pulmonic valve was what provided the substrate for the development of IE. To the authors’ knowledge, this is the first reported case of sub-pulmonic valve endocarditis in a patient with a history of modified Konno procedure.

Typically, right-sided endocarditis is conservatively managed with intravenous antibiotics in the absence of complications requiring surgical intervention. Surgical indications include persistent infection unresponsive to antibiotic therapy beyond two weeks, recurrent septic pulmonary emboli, septic shock, an increase in vegetation size >1 cm despite antibiotic therapy, new-onset or worsening renal and/or hepatic failure, worsening tricuspid regurgitation leading to right heart failure, and secondary multi-valvular involvement [10].

## Conclusions

We report a case of isolated sub-pulmonic valve endocarditis in a patient with a history of modified Kono procedure and a mechanical aortic valve. Pulmonic valve endocarditis is often difficult to diagnose given the challenge of imaging the pulmonic valve, and a low index of suspicion based on epidemiological data that favors left-sided valvular involvement in the majority of the cases of infective endocarditis. In patients with risk factors such as congenital heart disease, clinicians should employ a high index of suspicion and adequately image the pulmonic valve. The presence of septic embolization to the lungs should raise suspicion of right-sided endocarditis. Although there are no current guidelines regarding therapy, in patients without complications necessitating surgical intervention, an extended course of intravenous antibiotics is curative, as was the case with our patient.
